# Metroplasty increases the take-home baby rate by reducing pregnancy loss without changing the chance of conception in women with septate uterus: a retrospective, single-center, observational study

**DOI:** 10.1186/s12884-023-06191-3

**Published:** 2023-12-14

**Authors:** Akiko Omoto, Hiroshi Ishikawa, Mariko Inoue, Sachi Morimoto, Kaori Koga, Makio Shozu

**Affiliations:** 1 Department of Obstetrics and Gynecology, Graduate School of Medicine, Chiba University, 1-8-1, Inohana, Chuo-ku, Chiba, 260-8670 Japan; 2 Department of Obstetrics and Gynecology, Chiba Kaihin Municipal Hospital, Chiba, 261- 0012 Japan; 3 Department of Obstetrics and Gynecology, Matsudo City General Hospital, Matsudo, 270- 2296 Japan; 4 Evolution and Reproductive Biology, Medical Mycology Research Center, Chiba University, Chiba, 260-8673 Japan

**Keywords:** Miscarriage, Hysteroscopy, Septate uterus, Plastic surgery procedures, Pregnancy outcome

## Abstract

**Background:**

Although abdominal or hysteroscopic metroplasty for septate uterus is considered to reduce pregnancy loss and increase the take-home baby (THB) rate in women with a history of recurrent pregnancy loss, there exists an inherent risk of impaired fertility. This study aimed to clarify the reproductive outcomes of women with septate uterus who underwent abdominal and hysteroscopic metroplasty in a single center.

**Methods:**

This retrospective observational study enrolled 27 women who underwent metroplasty between 2007 and 2019. The analysis included women with septate uterus [European Society of Human Reproduction and Embryology (ESHRE)/European Society for Gynaecological Endoscopy (ESGE) type U2)] or septate-bicornuate uterus (ESHRE/ESGE type U3b) who underwent either abdominal or hysteroscopic metroplasty. Women who did not have an immediate desire to conceive were excluded from the analysis. As a rule, we recommended pregnancy without surgery for women who had not experienced repeated pregnancy loss. Abdominal metroplasty (ABM) was performed using the modified Tompkins’ method and hysteroscopic metroplasty was performed using hysteroscopic transcervical resection of the septum [transcervical metroplasty (TCM)]. The conception ratio was calculated as the number of women who achieved ≥ 1 conception/total number of women, the pregnancy loss ratio was calculated as the number of women who experienced ≥ 1 pregnancy loss/the number of women who conceived, and the THB ratio was calculated as the number of women who achieved ≥ 1 THB/total number of women.

**Results:**

Seventeen women underwent ABM and 10 women underwent TCM. Thirty-three conceptions and 26 babies were taken home after surgery. ABM did not change the ≥ 1 conception ratio (76% vs. 83% before and after surgery, respectively; RR = 1.08, *p* = 0.80). Meanwhile, ABM decreased the ≥ 1 pregnancy loss ratio (100% vs. 36%, RR = 0.36, *p* < 0.001) and increased the ≥ 1 THB ratio (12% vs. 71%, RR = 6.00, *p* < 0.01). Similarly, TCM did not change the ≥ 1 conception ratio, decreased the ≥ 1 pregnancy loss ratio, and increased the ≥ 1 THB ratio.

**Conclusions:**

Both abdominal and hysteroscopic metroplasty for septate uterus increased the THB rate by preventing pregnancy loss without affecting the chance of pregnancy.

**Trial registration:**

Not applicable.

**Supplementary Information:**

The online version contains supplementary material available at 10.1186/s12884-023-06191-3.

## Background

Congenital uterine anomalies impair pregnancy outcomes, increase the frequency of early miscarriage and preterm birth, and eventually decrease the live birth rate [1–4]. Recent systematic reviews and meta-analyses have revealed that these anomalies are associated with a higher risk of preterm birth, prelabor rupture of membranes, fetal malpresentation, fetal growth restriction, placental abruption, placenta previa, and cesarean birth compared with those without anomalies, and women with congenital uterine anomalies had a lower rate of live births and higher rate of first and second trimester miscarriage [5, 6]. Of these anomalies, septate uterus is defined as a uterus with a normal outline and an internal indentation at the fundal midline exceeding 50% of the uterine wall thickness. Bicorporeal-septate uterus is defined as a uterus with an abnormal fundal outline characterized by the presence of an absorption defect in addition to the main fusion defect, corresponding to the European Society of Human Reproduction and Embryology (ESHRE)/European Society for Gynaecological Endoscopy (ESGE) classification of uterine anomalies class U2a, U2b, and U3 [7]. The septate and bicorporeal-septate uteri have been consistently shown to impair pregnancy outcomes [5, 8].

Metroplasty is performed to improve the reproductive outcomes in women with septate uterus who have experienced adverse obstetric events, including early stage miscarriage, preterm birth, and subfertility. Hysteroscopic metroplasty, also called transcervical metroplasty (TCM), and open abdominal metroplasty (ABM) are performed to correct the shape of the uterine cavity. ABM is a conventional procedures performed using the the Strassmann, Tompkins, and Jones procedures, and their modifications [9–11]. Retrospective studies performed till the early 1990s have indicated that women with septate or bicorunuate uterus who underwent ABM have improved reproductive outcomes [12, 13]; however, ABM is highly invasive since it is an open surgery that enatils connection of the two split halves of the uterus into a single cavity. Concerns exist about peritoneal adhesion caused by abdominal surgery, which may lead to a new problem that impairs fertility [14]. Therefore, the therapeutic efficacy of ABM should be determined by balancing its protective effect against pregnancy loss and its detrimental impact on fertility and physical health. In addition, passing on the skills and knowlege about ABM to the next generation of surgeons is challenging because of the complexity of the technique and rarity of the procedure. In contrast, TCM is less invasive than ABM, and has become safer and more accessible with the development of resectoscopy equipment, allowing for easier implementation by gynecologists [15]. TCM reduces the risk of miscarriage and frequency of fetal malpresentation during the subsequent pregnancy in women with a complete or partial uterine septum [16]. In contrast, TCM has drawbacks such as intraoperative perforation of the uterus, which occurs in 1–4% of cases, and late-onset uterine rupture, which can occur during pregnancy or at delivery in 0.03% of women [17]. Spontaneous rupture of the uterus during pregnancy, albeit rare, could be life-threatening for both the mother and fetus, necessitating hysterectomy, which destroys fertility. Moreover, the TCM procedure cannot be used to unify the separated cavities into one simple cavity when the septate-cornuate uterus has a deep concavity at the uterine fundus. Thus, the application of TCM is limited in patients with septate-cornuate uterus, for which ABM using myometrial suture is indicated [18].

In the current TCM dominant era, we have performed both TCM and ABM in accordance with stringent inclusion and exclusion criteria. We deemed it important to elucidate the efficacy of ABM in women with septate-cornuate uteus who have experienced recurrent pregnancy loss, by performing both TCM and ABM at a single institution. Herein, we evaluated the pregnancy outcomes of TCM and ABM for septate or septate-bicornuate uterus in our facility. Moreover, we ascertained whether the treatment benefits can offset the adverse effects of surgery using the traditionally employed cumulative take-home baby (THB, defined as the birth of a live, healthy baby beyond 22 weeks of gestation who can be discharged home with the parents without significant health issues) rate and the per-cycle pregnancy loss rate, in addition to the conventionally used pregnancy loss rate.

## Methods

### Ethical approval

This retrospective observational study was conducted at the Department of Obstetrics and Gynecology, Chiba University Hospital, in collaboration with the Department of Obstetrics and Gynecology, Graduate School of Medicine, Chiba University, Chiba, Japan under approval of the Institutional Review Board (IRB) of the Chiba University Graduate School of Medicine (#M10613, approved on April 7, 2023). The IRB of the Chiba University Graduate School of Medicine waived the need of informed consent and the opt-out method was used to obtain consent from the participants. The study was conducted in accordance with the principles of the Declaration of Helsinki.

### Study design

We reviewed the clinical charts of women who were referred to Chiba University Hospital and underwent either ABM or TCM between January 1, 2007, and December 31, 2019. The combination of ultrasonography, hysterosalpingography, and magnetic resonance imaging (MRI) was used to assess the uterine shape. Women who had a uterine septum and met the eligibility criteria were included in this study.

The inclusion criteria for this study were as follows: (1) congenital uterine anomalies with a uterine septum that occupies more than half of the long axis of the uterine cavity corresponding to the ESHRE/ESGE classification of uterine anomalies class U2a, U2b, and U3c [7]; (2) no apparent cause of pregnancy loss other than the uterine anomaly; and (3) history of ≥ 3 miscarriages, or ≤ 2 miscarriages and unwilling to try the next conception without surgery. The reasons for unwillingness included advanced maternal age, long history of infertility, and extreme uterine anomalies such as U1a, which is highly suspicious of a high risk of pregnancy loss [7].

Women whose complete uterine septum had ruptured at the antecedent pregnancy, leading to a narrow opening between the right and left uterine cavities, were excluded from the analysis. We proposed immediate TCM instead of trial pregnancy without surgery, irrespective of the outcome of the first baby, because of the supposedly high risk of pregnancy loss. Women who did not wish to conceive immediately after ABM were excluded from the analysis. Consequently, one patient who underwent ABM and four patients who underwent TCM were excluded.

### Choice of the mode of surgery

The shape of the endometrial cavity, the previous pregnancy outcomes, and our own clinical experiences were taken into consideration, and the mode of surgery was chosen in conjunction with the patient after discussion.

**Fig. 1:**
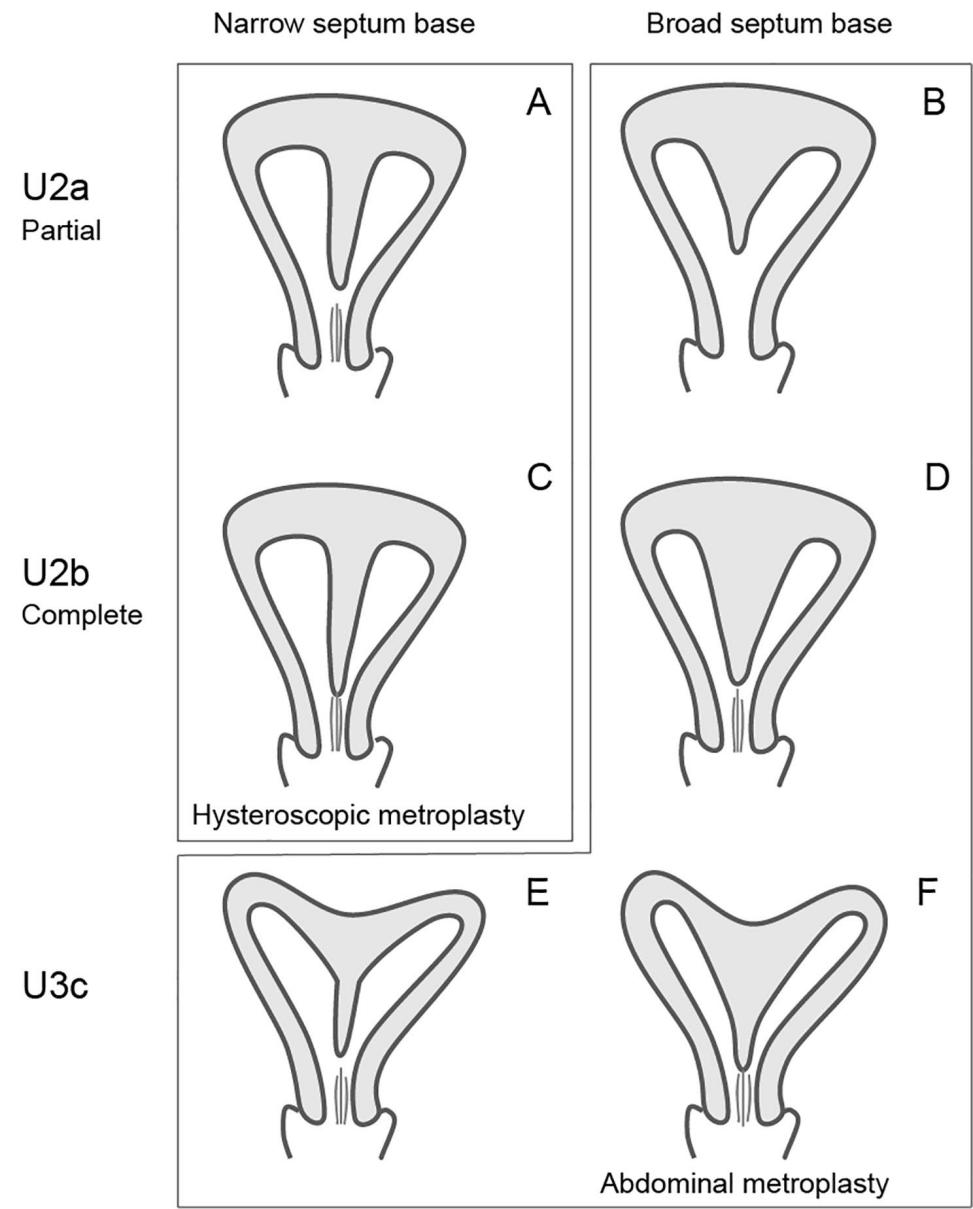
Definition of uterine shape in this study. The shapes of the septate uteri were classified using the ESHRE/ESGE classification for uterine anomalies and further classified based on the narrowness or broadness of the septum. **(A) (B)** Septate uterus with a partial septum classified as U2a **(C)(D)** Septate uterus with a complete septum classified as U2b **(E)(F)** Bicorporeal septate uterus classified as U3c. The uterus in the left row has a narrow septum **(A, C, and E)**. In contrast, the uterus in the right row has a broad septum **(B, D, and F)**. We preferred hysteroscopic metroplasty (transcervical metroplasty, TCM) for the shapes depicted in panels **A** and **C**, and abdominal metroplasty (ABM) for the shapes shown in panels B, **D, E, and F**. ESHRE: European Society of Human Reproduction and Embryology, ESGE: European Society for Gynaecological Endoscopy

Septate uterus with a partial or complete septum, which is often thin, and associated with a flat or convex fundus that was classified as Class U2a and U2b was considered for TCM (Fig. 1A and C). Even if the uterine shape was classified as Class U2a and U2b, a thick and broad septum was considered an indication of ABM (Fig. 1B and D). A uterus with a septum associated with a deep concavity at its fundus that was classified as Class U3c was considered for ABM (Fig. 1E and F). In cases of intermediate morphology or complications with other types of anomalies (such as Class Ia), the choice of ABM or TCM was based on the degree to which the reconstructed uterine cavity would approximate the average after hysteroscopic resection of the septum without myometrial reconstruction.

### Outcomes measures

The conception rate, spontaneous abortion rate, and THB rate were compared before and after metroplasty. THB is defined as the birth of a live, healthy baby beyond 22 weeks of gestation who can be discharged home with the parents without significant health issues. A clinical pregnancy was diagnosed on the basis of identification of a gestational sac. Artificial abortion after surgery was not counted as a conception outcome. The secondary endpoints were the time to the initial pregnancy after metroplasty, time to THB, and postoperative complications that prolonged hospital stay.

### Surgical procedure for ABM (Fig. 2)

**Fig. 2:**
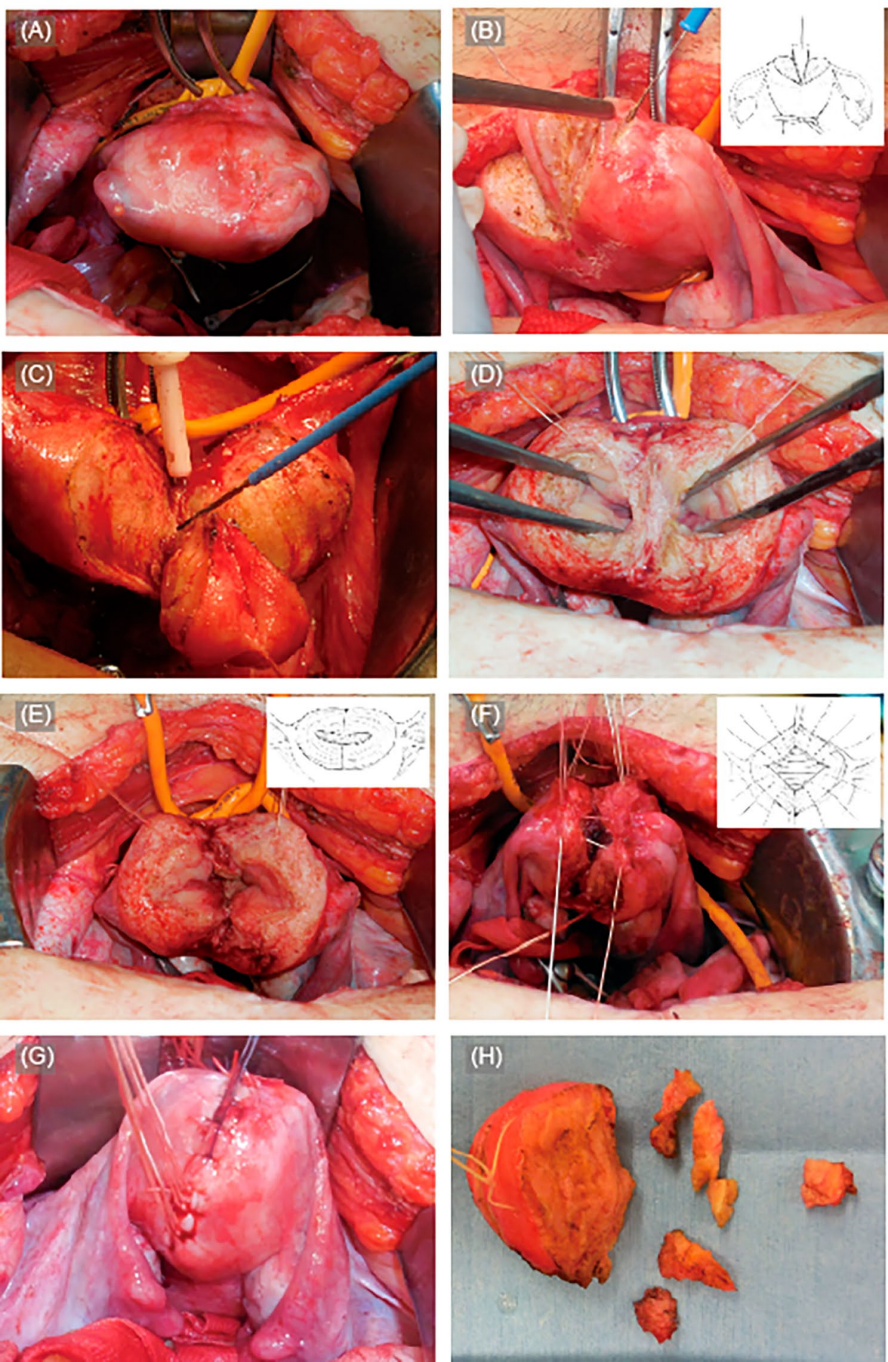
Procedures for abdominal metroplasty (ABM). Representative operative findings of ABM are shown. Our procedure entailed the modified Tompkins’ method, followed by Strassman’s method. **(A)** Occlusion of blood flow to the uterine body was achieved using tourniquets and clipping of the ovarian vessels **(B)(C)** Removal of the uterine septum using wedge resection from outside the uterine fundus to transform a uterus into a bicornuate uterus **(D)** Opening of both cavities of the bicornuate uterus **(E)** Incision of the remaining septum in the longitudinal direction (left to right) to open the cavities of the two horns **(F)** Suturing of the myometrial layer in the anterior-posterior direction **(G)** Completion of myometrial suturing, and releasing the tourniquet and unclipping the ovarian vessels **(H)** Septum tissues excised during the ABM. The thin septum was incised along the coronal plane using an electrocautery electrode

One researcher (M.S.) performed ABM throughout the study period using the modified Tompkins’ method, followed by Strassman’s method, as described elsewhere [18, 19]. After occlusion of blood flow to the uterine body using a tourniquet and clipping of the ovarian vessels (Fig. 2A), the uterine septum was removed by wedge resection from outside the uterine fundus to transform the uterus into a bicornuate uterus (Fig. 2B and C). Thereafter, the medial side of the “bicornuate uterus” was incised in the longitudinal direction (left to right) to open the cavities of the two horns (Fig. 2D and E), the myometrium of which was sewn in the anterior-posterior direction to unite the right and left cavities into one using the Strassman maneuver (Fig. 2F and G). If the septum was located at the uterine cervix, the septum of the uterine cervix was preserved. The septum was removed thoroughly to ensure that the remaining thickness of the medial side of the muscle layer for each of the uterine horns was approximately 3 mm or less, so that the reconstructed uterine fundus would not budge again. When a thin septum remained on the caudal side of the neocavity, the septum was incised along the coronal plane using an electrocautery electrode up to the internal os, with or without the application of an electric current using hysteroscopic surgery (Fig. 2H).

**Fig. 3:**
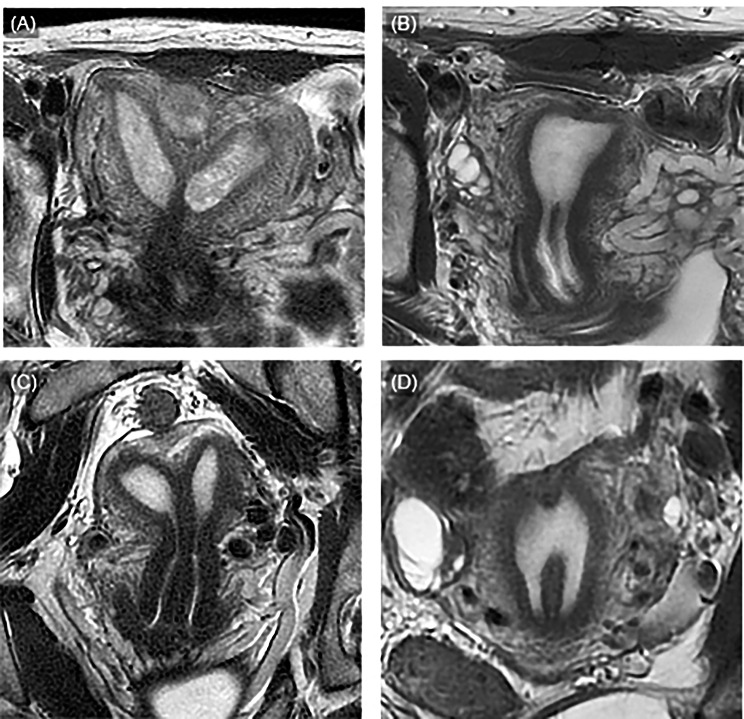
Imaging studies before and after ABM. Three cases are shown before and after ABM. **(A)** T2-weighed MRI of a septate uterus before metroplasty. The uterine shape was ESHRE/ESGE type U2a. **(B)** T2-weighed MRI after metroplasty for the case depicted in panel A. **(C)** T2-weighed MRI image before metroplasty. The uterine shape was ESHRE/ESGE type U2a. **(D)** T2-weighed MRI after metroplasty in the case depicted in panel C. MRI: magnetic resonance imaging, ESHRE: European Society of Human Reproduction and Embryology, ESGE: European Society for Gynaecological Endoscopy

Imaging studies, including ultrasonography, MRI, and hysterosalpingography, were performed 3–4 months after metroplasty to evaluate the uterine morphology (Fig. 3). Hysteroscopy was performed to evaluate the endometrial conditions before conception. Additional hysteroscopic surgery was considered if endometrial synechia occurred or septal resection was insufficient.

### Statistical analysis

Statistical analysis was performed using EZR ver1.54 and JMP version 14. The Fisher’s exact probability test and Wilcoxon’s signed-rank test were used to evaluate categorical data and continuous variables, respectively. P-values < 0.05 were considered statistically significant. The log-rank test was used to compare the duration required to achieve the outcomes between the subgroups. The chi-squared test was used to assess the independence of conception before and after surgery.

## Results

Twenty-seven patients were included in the analysis: 17 women underwent ABM, and 10 underwent TCM. Two women who underwent ABM had an intramuscular fibroid at the septum base, which was removed during metroplasty. Representative operative findings of ABM and imaging studies before and after metroplasty are shown in Figs. 2 and 3.

The patients’ median age was 33 years. 74% of patients presented with pregnancy loss, and the remaining presented with infertility (Table 1). The age distribution and cause of hospital visits did not differ between the ABM and TCM groups. However, the frequency of previous pregnancy loss was significantly higher in women who underwent ABM than in those who underwent TCM (*p* < 0.05), even if the analysis was limited to women who had presented with pregnancy loss. Moreover, the frequency of U3 anomalies was higher and that of U2 anomalies was lower in women who underwent ABM compared to those who underwent TCM (*p* = 0.06). Consequently, ABM was performed more frequently for women with more pregnancy loss and a greater degree of uterine concavity. The follow-up period for the pregnancy outcome was more prolonged in women who underwent ABM than in those who underwent TCM (*p* = 0.07).

**Table 1:** Participants’ clinical characteristics

		Abdominal metroplasty (ABM)		Transcervical metroplasty (TCM)		*p* value^$^	Total	
		n (%)	Median (range)	n (%)	Median (range)		n (%)	Median (range)
Total number of women (n)		17		10				
Age (years)	Median (range)		32.5 (23–39)		33 (25–40)	n.s.		33 (13–40)
	35< (n, % total)	7 (41%)		3 (30%)		n.s.	10 (37%)	
	≤ 35 (n, % total)	10 (59%)		7 (70%)			17 (63%)	
Presenting complaint	Pregnancy loss (n)	13 (76%)		7 (70%)		n.s.	20 (74%)	
	Infertility (n)	4 (24%)		3 (30%)			7 (26%)	
Number of previous pregnancy losses per woman, including those without an experience of pregnancy	Median (range)		2 (0–12)		1 (0–2)	< 0.05		1 (0–12)
Number of previous pregnancy loss per woman who had experienced ≥ 1 pregnancy loss	Median (range)		2 (1–12)		1 (1–2)	< 0.05		1.5 (1–12)
Uterine shape^#^	U2 (n, % total)	9 (53%)		9 (90%)		0.06	18 (67%)	
	U3 (n, % total)	8 (47%)		1 (10%)			9 (33%)	
Follow-up period after surgery (months)	Median (range)		19 (5-103)		9.5 (3–37)	0.07		12 (3-103)

n.s.: not statistically significant

#Uterine shape corresponds to the 2013 ESHRE/ESGE classification of uterine anomalies [7]

$The test for continuous variables was conducted using Fisher’s exact probability test and that for categorical variables was conducted using Wilcoxon’s signed-rank test

### Per conception analysis

**Fig. 4:**
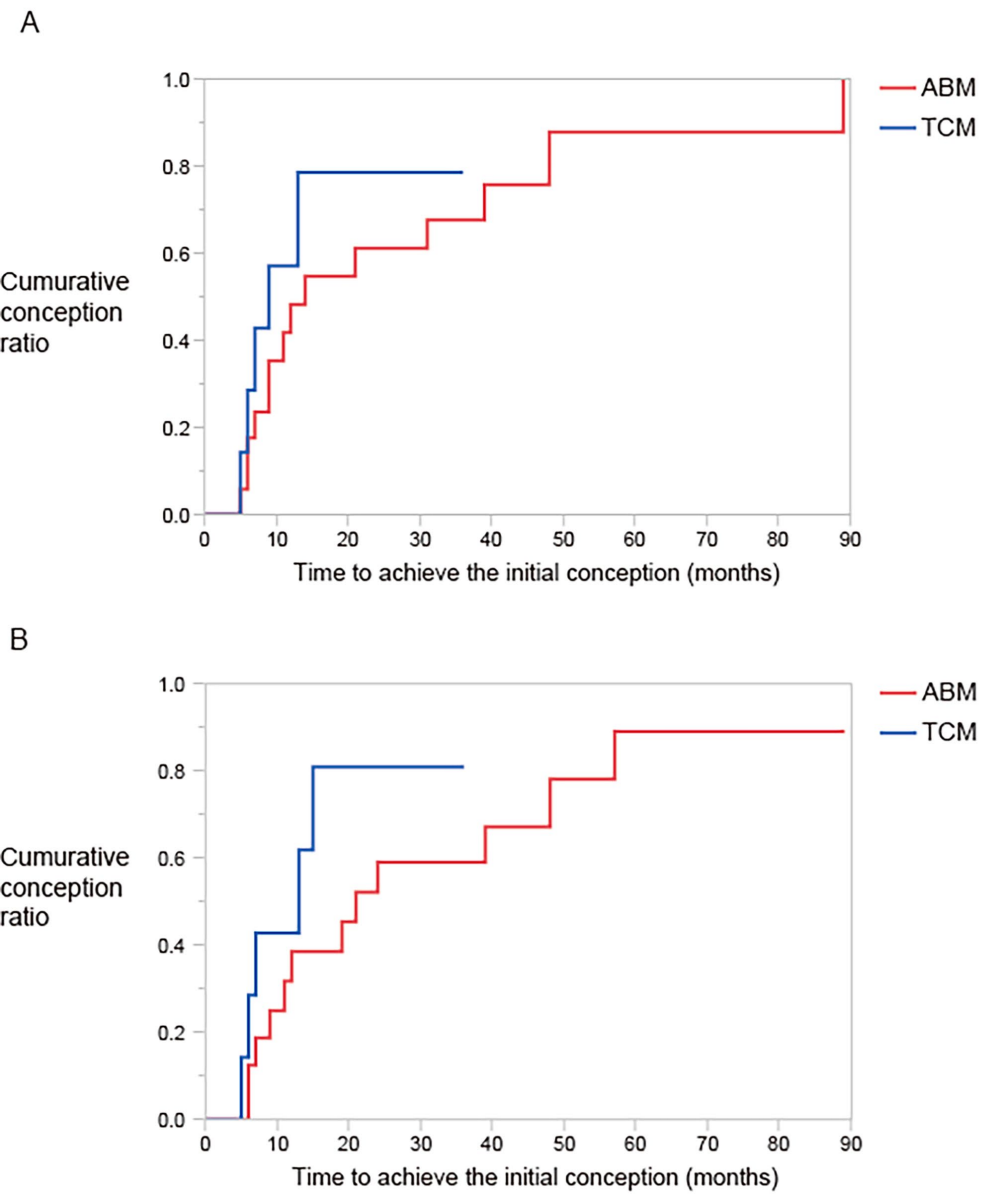
Cumulative conception rate after metroplasty. **(A)** Cumulative incident rate for the initial pregnancy **(B)** Cumulative incident rate for the initial pregnancy yielding a take-home baby. ABM: abdominal metroplasty, TCM: transcervical metroplasty

Overall, 33 conceptions were achieved in 27 women after metroplasty (Table 2). The number of conceptions per patient did not differ between the ABM and TCM groups (mean ± SEM: 1.3 ± 0.2 and 1.1 ± 0.3, respectively, *p* = 0.49). The time to achieve first conception after metroplasty was more prolonged in women who underwent ABM than in those who underwent TCM, but the difference was not statistically significant (median, 14 months vs. 8 months, *p* = 0.27, log-rank test) (Fig. 4A).

**Table 2:** Conception and pregnancy loss before and after metroplasty

	Abdominal metroplasty (ABM)				Transcervical metroplasty (TCM)				Total			
Outcomes	Pre-surgery	Post-surgery	RR^$^ [95% CI]	*p* value^$^	Pre-surgery	Post-surgery	RR^$^ [95% CI]	*p* value^$^	Pre-surgery	Post-surgery	RR^$^ [95% CI]	*p* value^$^
Total number of women (n)	17	17			10	10			27	27		
*Per conception analysis*												
Total number of conception (% conception)	42 (100%)	22 (100%)			8 (100%)	11 (100%)			50 (100%)	33 (100%)		
Pregnancy loss (% conception)	39 (93%)	6 (27%)	0.29 [0.15–0.58]	< 0.0001	8 (100%)	1 (9%)	0.09 [0.01–0.59]	< 0.0001	47 (94%)	7 (21%)	0.23 [0.12–0.44]	< 0.0001
Miscarriage (< 22 weeks)	36	5			6	1			42	6		
Late pregnancy loss (≥ 22 weeks)	3	1			2	0			5	1		
Take-home baby (% conception)	3 (7%)	16 (72%)	10.18 [3.32–31.20]	< 0.0001	0 (0%)	10 (91%)	> 10.00	< 0.0001	3 (6%)	26 (79%)	13.13 [4.32–39.90]	< 0.0001
First baby	2	12			0	6			2	18		
Second baby	1	4			0	3			1	7		
Third baby	0	0			0	1			0	1		
*Per patient analysis*												
Women with ≥ 1 conception (% total women)	13 (76%)	14 (83%)	1.08 [0.76–1.52]	n.s.	7 (70%)	6 (60%)	0.86 [0.45–1.34]	n.s	20 (74%)	20 (74%)	1.00 [0.73–1.37]	n.s.
Women with pregnancy loss (per conceived women)	13 (100%)	5 (36%)	0.36 [0.18–0.72]	< 0.001	7 (100%)	1 (17%)	0.17 [0.03-1.00]	< 0.01	20 (100%)	6 (30%)	0.30 [0.15–0.59]	< 0.0001
Women with ≤ 21-week miscarriage (% conceived women)	12 (92%)	4 (29%)	0.31 [0.13–0.72]	< 0.01	5 (50%)	1 (10%)	0.20 [0.03–1.42]	n.s	17 (85%)	5 (25%)	0.29 [0.13–0.64]	< 0.001
Women with ≥ 22-week pregnancy loss (% conceived women)	3 (23%)	1 (7%)	0.31 [0.04–2.61]	n.s.	2 (20%)	0 (0%)	0.00	n.s	5 (25%)	1 (5%)	0.20 [0.02–1.56]	n.s.
Women with ≥ 22-week pregnancy (% conceived women)	4 (31%)	13 (93%)	3.02 [1.32–6.91]	< 0.01	2 (29%)	6 (100%)	3.50 [1.08–11.29]	< 0.05	6 (30%)	19 (95%)	3.17 [1.61–6.23]	< 0.0001
Women with ≥ 1 take-home baby (% total women)	2 (12%)	12 (71%)	6.00 [1.58–22.86]	< 0.01	0 (0%)	6 (60%)	≥ 10.00	< 0.01	2 (7%)	18 (67%)	9.00 [2.31–35.07]	< 0.0001

* All women with or without an experience of pregnancy loss were included

# Only women who had experienced ≧ 1 pregnancy loss were included

$The independence of conception before and after surgery was evaluated using the chi-square test

RR, relative risk; 95% CI, 95% confidence interval

Seven of 33 postsurgical pregnancies (21%) ended in pregnancy loss, and the relative risk (RR) of pregnancy loss decreased to 0.23 (*p* < 0.0001) after metroplasty (Table 2). Both ABM and TCM reduced pregnancy loss; however, the RR remained higher in women who underwent ABM than in those who underwent TCM (RR, 0.29 vs. 0.09, respectively, *p* < 0.19) (Table 2). Subgroup analysis of the background factors revealed that the risk of pregnancy loss remained high after ABM for U2 anomaly, whereas it was nullified in U3 anomaly (RR, 0.52 vs. 0.00, respectively, *p* < 0.05, χ^2^ test) (Supplemental Table 1). Thus, ABM is ineffective for U2 anomaly and highly effective for U3 anomaly.

The causes of postsurgical pregnancy loss were estimated in four cases as follows: two cases of fetal trisomy, one intrauterine infection (chorioamnionitis), and one case of early-onset eclampsia (Table 3). Twenty-six babies of 18 women were taken home after surgery, including 7 s babies and 1 third baby (Table 2). The THB rate per conception increased from 6 to 79% after surgery (RR: 13.13).

**Table 3:** Estimated causes of postsurgical pregnancy loss

Uterine shape^#^	Metroplasty procedure	Gestational age at pregnancy loss (weeks)	Estimated causes of pregnancy loss or related clinical events
U2b	ABM	5	Spontaneous abortion. Chromosome analysis was not performed.
U2b	ABM	5	Spontaneous abortion. Chromosome analysis was not performed.
U2b	ABM	6	Trisomy (chromosome 12)
U2a	ABM	7	Trisomy (chromosome 10)
U2b	TCM	8	Spontaneous abortion after cardiac arrest at 7 weeks. Chromosome analysis was not performed.
U2a	ABM	21	Intrauterine infection
U2a	ABM	24	Elective cesarean section due to eclampsia associated with hypertension
U2b	ABM	5	Spontaneous abortion. Chromosome analysis was not performed.
U2b	ABM	5	Spontaneous abortion. Chromosome analysis was not performed.

#Uterine shape corresponds to the 2013 ESHRE/ESGE classification of uterine anomalies [7]

ABM, abdominal metroplasty; TCM, transcervical metroplasty

### Per patient analysis

The censored conception rate (number of women who achieved ≥ 1 pregnancy/total number of women) did not differ before and after metroplasty (74% for both), nor did it differ between ABM and TCM (76% vs. 70% and 83% vs. 60% before and after metroplasty, respectively) (Table 2). The time to first conception was longer in women who underwent ABM than in those who underwent TCM, but the difference was not statistically significant (median: 21 months vs. one month, *p* = 0.33, log-rank test) (Fig. 4B). The RR for ≥ 1 pregnancy loss decreased after metroplasty (RR, 0.30, *p* < 0.0001) (Table 2).

After surgery, 18 of 27 women (67%) achieved ≥ 1 THB during the follow-up period (RR, 9.00; Table 2). This increase was not due to an increase in the pregnancy rate but a decrease in pregnancy loss, as described above. Subgroup analysis for ABM showed that the presence of U3 anomaly (RR, 0) and maternal age < 35 years (RR, 0.13) made greater contributions toward reducing pregnancy loss and achieving ≥ 1 THB (Supplementary Table 2). The time to the first THB was longer in women who underwent ABM than in those who underwent TCM, but the difference was insignificant (median: 21 months vs. 13 months, *p* = 0.34, log-rank test) (Fig. 4A).

### Postsurgical complications

Postsurgical endometrial adhesions (synechia) occurred in one woman who underwent ABM. Thereafter, she underwent hysteroscopic synechiolysis and achieved a THB. All 16 pregnancies after ABM continued beyond 24 weeks and were terminated by elective cesarean section. Of the 6 women who conceived after TCM, five delivered vaginally, and one woman delivered by cesarean section for obstetric reasons. There was no uterine rupture during pregnancy or labor. Placenta accrete occurred in two women who underwent ABM: one underwent cesarean hysterectomy, and the other underwent manual removal of the placenta accrete and conceived a second baby later.

## Discussion

This study demonstrated that metroplasty for septate uterus increased the THB rate by preventing pregnancy loss without affecting the chance of conception. Although ABM was effective in both the septate uterus and septate-bicornuate uterus, it was more effective in the latter than the former. One patient needed a cesarean hysterectomy due to placenta increta (5% of total conceptions). Otherwise, no severe complications occurred. Thus, ABM is a treatment option for septate-bicornate uterus.

In this study, we demonstrated the efficacy of metroplasty by comparing the pregnancy outcomes before and after metroplasty. Several studies have employed a similar design to depict the utility of metroplasty. Patton et al. treated 16 septate uteri with duplicated cervix using ABM (modified Tompkins, n = 5) or TCM (n = 11). Although the pre-surgical obstetrical history was not fully available in their study, metroplasty decreased the pregnancy loss rate from 81% (13/16 conceptions for 9 women) to 18% (3/17 conceptions for 14 women), meaning that the live birth rate increased (including ongoing third-trimester pregnancy) from 19–82%. Ayhan et al. reported the pregnancy outcomes of ABM (Tompkins’ or Jones and Jones’ operation) for 49 women with septate uterus: the live birth rate increased from 4% (7/173 conception) to 65% (30/46 conceptions) after metroplasty [12]. Similarly, Helm et al. reported that ABM (Tompkins’ or Jones and Jones’ operation) increased the live birth rate from 4% (2/44 conceptions) to 76% (16/21 conceptions) in 17 women with septate uterus [11]. Kessler et al. performed ABM (Tomkins’, Jones and Jones’ operation, or Strassman’s operation) in 17 patients with septate uterus, bicornuate uterus, or both and found that the live birth rate increased from 14% (6/42 conceptions) to 85% (23/27) [10]. All of these studies yielded concurrent findings that metroplasty increases the live birth rate in pregnant women with septate- and septate-bicornuate uterus from 20% or less to 65% or higher, although the classification of the uterine anomaly, indications of surgical intervention, and surgical technique may have differed slightly among them. The results of our study obtained using the cumulative THB ratio instead of the live birth ratio are also consistent with those of previous studies. As we could not determine whether the pregnancy loss was due to a uterine anomaly or other causes, such as chromosomal anomaly, we simply compared the number of total pregnancy losses and the number of THB without any validation. Consequently, the crude pregnancy loss rate was 21% after surgery, equivalent to that of 33-year-old women in the general population [19]. We wish to emphasize the necessity of ABM in the era dominated by hysteroscopic surgery. The indications for ABM and TCM were quite distinct in our facility: we performed ABM in cases where TCM would not be facilitate sufficient reconstruction of the uterine cavity and found that ABM was effective in preventing pregnancy loss in U3 anomalies.

We observed that ABM was less effective in preventing pregnancy loss and yielding a lower THB outcome than TCM (the RR for pregnancy loss was 0.29 and 0.09 for ABM and TCM, respectively). Subgroup analysis revealed that the low therapeutic efficacy was primarily due to the ineffectiveness of ABM in treating U2 anomalies. There are three explanations for this. First, the ABM procedure was insufficient for functional recovery of the U2 anomaly. The second was patient selection bias: in U2 anomalies, the broader the septal base (i.e., the greater the severity of the anomaly), the greater the tendency to allocate the patient to ABM. The third explanation is an alpha error: we detected the difference by chance. The review of individual cases showed that at least three pregnancy losses were not caused directly by a uterine anomaly, as there were two chromosomal trisomy cases and one case of early-onset preeclampsia.

In addition to per-conception analysis, we conducted per-patient analysis where individual data were censored at the first THB. It is possible that the use of the cumulative THB rate eliminated the over-contribution of “fertile” women who repeatedly conceived after surgery and distorted the statistical analysis. Using per-patient analysis, we demonstrated with greater clarity that surgery increased the THB rate not by enhancing the chance of conception but by preventing pregnancy loss.

A strength of this study is that the same physician performed the metroplasty using the same technique throughout the study period. This ensured consistency of the surgical techniques for TCM and ABM, eliminating the effect of disparities between operators and facilities. Moreover, the efficacy of ABM was not overestimated due to selection bias because the clinical background characteristics of the ABM group tended to be more severe than those of the TCM group. In contrast, the single-center setting and performance of the procedures by a solo surgeon may have potential limitations. Another limitation is the small sample size. Moreover, because this study lacks a control group in which we prospectively observes the pregnancy oucomes of new conception without surgical intervention in the control group. Thus, we cannot eliminate the possibility that the pregnancy outcomes could have improved without intervention. However, this is unlikely because the term delivery rate for women with uterine anomaly who conceived without surgery was 43%, approximatly a half that of women without uterine anomaly [1]. We believe that the findings of this study, despite its limited sample size, are valuable for implementing narrative-based medicine tailored to individual patients.

## Conclusions

Both ABM and TCM for septate uterus reduce pregnancy loss without affecting the chance of pregnancy in women with septate uterus who have a history of recurrent pregnancy loss. ABM may be specifically effective for a septate-bicornuate uterus with a deeply concave uterine fundus. Considering that robotic and laparoscopic surgeries have entered the mainstream in the field of gynecologic surgery, the outcomes of ABM presented in the current study serve as a benchmark for future surgeries.

### Electronic supplementary material

Below is the link to the electronic supplementary material.

Supplementary Material 1

Supplementary Material 2

## Data Availability

The datasets generated and analyzed during the current study are not publicly available due to containing personal identification information but are available from the corresponding author on reasonable request.

## List of abbreviations

ABM: abdominal metroplasty
ESGE: European Society for Gynaecological Endoscopy
ESHRE: European Society of Human Reproduction and Embryology
MRI: magnetic resonance imaging
SEM: standard error of the mean
RR: relative risk
TCM: transcervical metroplasty
THB: take-home baby
